# Neural activity induced by visual food stimuli presented out of awareness: a preliminary magnetoencephalography study

**DOI:** 10.1038/s41598-018-21383-0

**Published:** 2018-02-15

**Authors:** Katsuko Takada, Akira Ishii, Takashi Matsuo, Chika Nakamura, Masato Uji, Takahiro Yoshikawa

**Affiliations:** Department of Sports Medicine, Osaka City University Graduate School of Medicine, 1-4-3 Asahimachi, Abeno-ku, Osaka, 545-8585 Japan

## Abstract

Obesity is a major public health problem in modern society. Appetitive behavior has been proposed to be partially driven by unconscious decision-making processes and thus, targeting the unconscious cognitive processes related to eating behavior is essential to develop strategies for overweight individuals and obese patients. Here, we presented food pictures below the threshold of awareness to healthy male volunteers and examined neural activity related to appetitive behavior using magnetoencephalography. We found that, among participants who did not recognize food pictures during the experiment, an index of heart rate variability assessed by electrocardiography (low-frequency component power/high-frequency component power ratio, LF/HF) just after picture presentation was increased compared with that just before presentation, and the increase in LF/HF was negatively associated with the score for cognitive restraint of food intake. In addition, increased LF/HF was negatively associated with increased alpha band power in Brodmann area (BA) 47 caused by food pictures presented below the threshold of awareness, and level of cognitive restraint was positively associated with increased alpha band power in BA13. Our findings may provide valuable clues to the development of methods assessing unconscious regulation of appetite and offer avenues for further study of the neural mechanisms related to eating behavior.

## Introduction

Obesity is a major public health problem in modern society. The prevalence of overweight individuals and obesity are increasing worldwide: The prevalence of overweight individuals and obesity in the adult population exceeds 60% in the United States and 50% in the United Kingdom^1^. Obesity is a cause of many health problems, including diabetes mellitus, dyslipidemia, hypertension, coronary heart disease, certain kinds of cancer such as colon cancer, and sleep-breathing disorders^2^.

Body weight is determined by the balance between energy expenditure and food intake: even a caloric intake less than 0.5% over energy expenditure can reportedly lead to weight gain^3,4^. Appetite is controlled by homeostatic and non-homeostatic mechanisms^5–7^. The hypothalamus, gut hormones, and adipokines are involved in the homeostatic control of appetite whereas the neural networks among the forebrain and brainstem, including reward-related brain regions, are involved in the non-homeostatic control of appetite. Particularly in humans, non-homeostatic control of appetite is thought to be an important factor that regulates food intake in terms of satiety^6^. It has been suggested that the abundance of food cues in the environment and the increased accessibility to palatable foods are related to excessive food intake through a variety of conscious and unconscious processes that result in an increasing prevalence of obesity in modern society. Among the unconscious processes that could have an effect is the non-homeostatic control of appetite^6,7^. Therefore, along with identifying the processes underlying homeostatic control of appetite, clarifying the neural mechanisms of non-homeostatic control of appetite, in particular, those of the food-related behavior induced by the food cues, is important.

Various reports have investigated the neural mechanisms of food-related behaviors through neural responses to visual food cues^8^. For example, a study using magnetoencephalography (MEG) reported that the intensity of the equivalent current dipole in the insular cortex evoked while viewing food pictures was associated with self-awareness of appetitive motives under fasting conditions^9^, and that neural activity in the insular cortex was reduced after consuming rice balls to the point before satiety^10^. Activation in the dorsolateral prefrontal cortex (DLPFC) and dorsal striatum correlated with the degree of dietary restraint in a functional magnetic resonance imaging (fMRI) study^11^ and a decrease in theta-band (4–8 Hz) power in the DLPFC was observed when participants were instructed to suppress appetitive motivation in an MEG study, suggesting that the DLPFC is involved in the suppression of motivation to eat^12^. In line with these findings, previous studies revealed that activation in the insular cortex caused by visual food cues was greater in obese individuals than in lean individuals^13–15^, and activation in the DLPFC caused by visual food cues was stronger in obese individuals than in lean participants^15^. These findings were interpreted as showing enhancement of appetitive motive and increased effort for appetite control in obese individuals.

Given such findings, evidence has been accumulating that the DLPFC and other brain regions involved in cognitive control are important for the intentional control of appetite. On the other hand, appetitive behavior has been proposed to be driven more by unconscious decision-making processes than by conscious processes^16^, and targeting the unconscious cognitive processes related to eating behavior is essential to develop strategies against overweight individuals and obesity^16,17^. Of course, much of the process of the homeostatic control of appetite is below the threshold of conscious awareness and there seems to be interactions between the processes of the homeostatic and non-homeostatic controls of appetite^18^. Thus, the process of the non-homeostatic control of appetite seems to include some unconscious processes related to appetite by nature^18^. As for the neural mechanisms of the food-related behaviors induced by visual food cues, there have been numerous studies which investigated the neural activities induced by viewing the food cues presented above the threshold of awareness^8^. However, as far as we know, there have been no reports directly investigated the neural mechanisms of the food-related behaviors induced by visual food cues presented below the threshold of conscious awareness. There is a possibility that these kind of neural mechanisms (i.e., the neural mechanisms related to the control of the appetite induced by visual food cues which affect food-related behaviors of individuals although they are not aware of the fact that the food cues have affected their behavior) play important roles in the unconscious decision-making process related to the food-related behavior.

The present study aimed to clarify the neural activity induced by visual food cues although individuals are not aware of the fact that they viewed the visual food cues. We used a visual backward masking procedure^19–21^ to present visual food cues below the threshold of awareness^22,23^ and recorded the neural activity observed during the presentation of the visual food cue using MEG to detect changes in oscillatory power reflecting changes in neural dynamics^24–26^. We hypothesized that the neural activity induced by viewing a visual food cue presented below the threshold of conscious awareness would reflect appetitive behaviors such as cognitive control of appetite if the unconscious control of appetite is essential as proposed and assessed the neural activity induced by viewing a visual food cue presented below the threshold of conscious awareness by using MEG. We also assessed the alterations in the measurements of electrocardiography (ECG) which would be caused by viewing the visual food cue presented below the threshold of conscious awareness to provide secondary evidence that the presentation of masked food pictures had some effects on neural systems.

## Materials and Methods

### Participants

Twenty healthy male volunteers (mean (±standard deviation, SD) age, 20.6 ± 2.3 years) participated in this study. All participants were confirmed to be right-handed according to a questionnaire^27^. Current cigarette smokers, individuals with a history of mental or neural and/or upper extremity disorder, and those taking medications that affect the activity of the central nervous system were excluded. The Ethics Committee of Osaka City University Graduate School of Medicine approved the protocol of this study (approval number, 3680). All participants provided written informed consent in accordance with the principles of the Declaration of Helsinki and the Ethical Guidelines for Medical and Health Research Involving Human Subjects in Japan (Ministry of Education, Culture, Sports, Science and Technology of Japan and Ministry of Health, Labor and Welfare of Japan). As a result, the MEG data from 14 participants were analyzed as described in the Results.

### Experimental design

For 1 day before the experiment, participants were instructed to finish dinner by 21:00, to fast overnight (drinking water was allowed), to avoid intense physical and mental activity, and to maintain normal sleeping hours.

**Figure 1:**
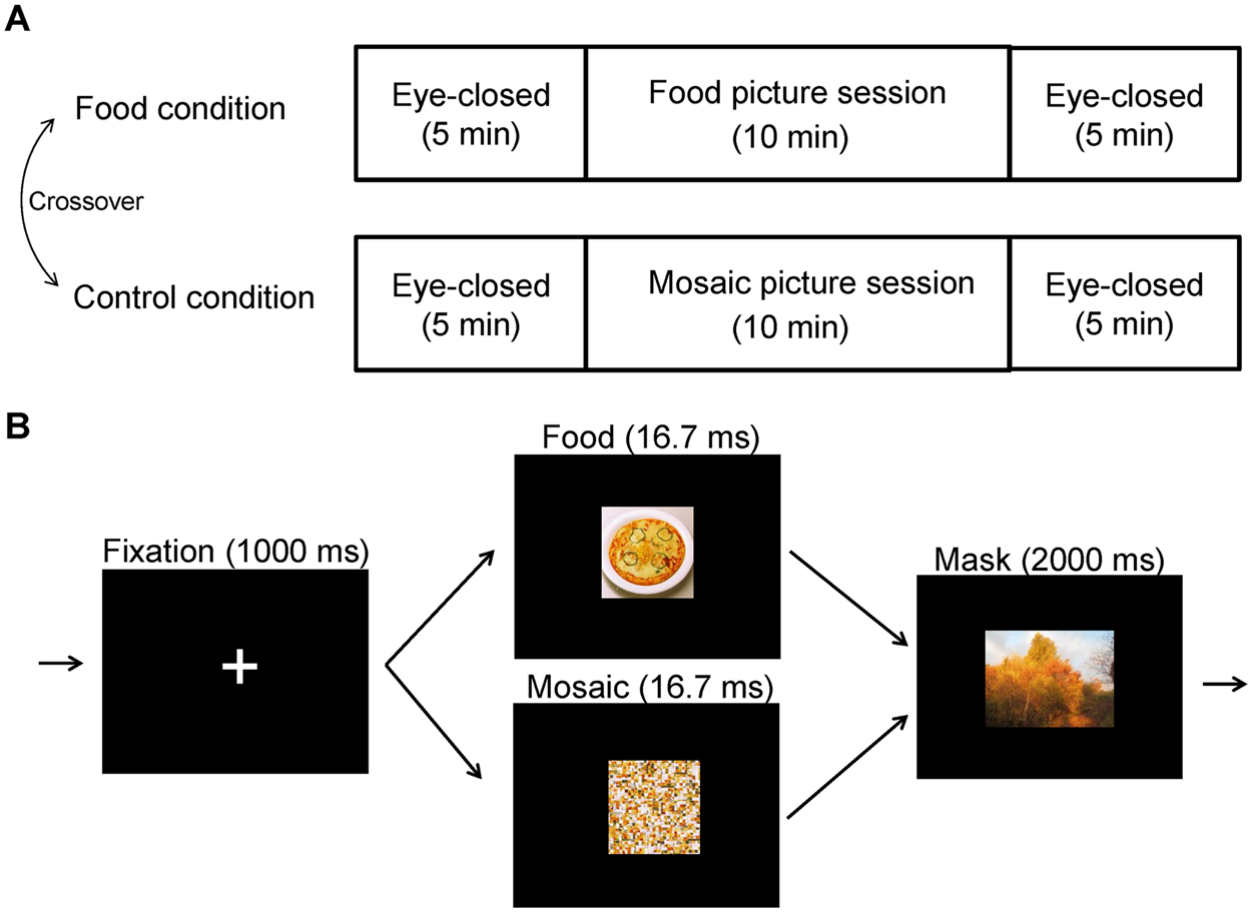
Experimental design. (**A**) The experiment consisted of food and control conditions, with each condition performed on the same day in a double-crossover fashion. In the food and control conditions, the participant lay in a supine position on a bed placed in a magnetically shielded room, and was asked to view a visual stimulus projected onto a screen placed in front of the eyes. (**B**) The visual stimuli used in the food condition consisted of a fixation cross, a food-picture, and a mask-picture. The visual stimulus used in the control condition consisted of a fixation cross, a mosaic-picture, and a mask-picture. These sequences of visual presentations were played 200 times in the food and control conditions, respectively. Twenty pictures of typical modern Japanese food items were used as food-pictures, and 20 pictures of non-food items such as scenery or buildings were used as mask-pictures. The mosaic pictures were created from the food-pictures used in the food condition to control for luminance and color between foods and control conditions. The permission to include the food-picture in this figure was obtained from Kagawa Nutrition University Publishing Division and the permission to include the mask-picture in this figure was obtained from the copyright holder of this image.

The experiment consisted of a food and a control conditions and each condition was performed on the same day in a double-crossover fashion (Fig. 1A). In the food and control conditions, the participant lay in a supine position on a bed placed in a magnetically shielded room and was asked to view a visual stimulus projected onto a screen placed in front of the participant using a projector (PG-B10S; SHARP, Osaka, Japan). The visual stimulus used in the food condition consisted of a fixation cross (for 1000 ms), a food-picture (16.7 ms), and a mask-picture (2000 ms) (Fig. 1B). This sequence of visual presentations was played 200 times. Twenty pictures of typical modern Japanese food items were used as food pictures^9,10,12,28^, and 20 pictures of non-food items such as scenery or buildings were used as mask-pictures: Each picture was used 10 times to construct the 200-picture set. The visual stimulus used in the control condition consisted of a fixation cross (for 1000 ms), a mosaic-picture (16.7 ms), and a mask-picture (2000 ms) (Fig. 1B). Mosaic pictures were created from the food-pictures used in the food condition using commercial software (Adobe Photoshop Elements 6.0; Adobe Systems, San Jose, CA) to control for luminance and color between food and control conditions^9,29,30^. The set of mask-pictures used in the control condition was the same as that used in the food condition. Neural activities caused by viewing visual stimuli in the food and control conditions were recorded using MEG. Just before and after the food-picture and mosaic-picture sessions in the food and control conditions, respectively, ECG was recorded while the participant lay on a bed quietly with eyes closed for 5 min. After the end of the experiment on day 2, the participant was asked to answer the Japanese version of the Three Factor Eating Questionnaire (TFEQ) Revised 21-item version, to assess eating behavior^31–33^. Just after the food and mosaic picture sessions, we asked our participants whether they could recognize any images other than the mask-pictures (i.e., the pictures of scenery and buildings) during the food and mosaic picture sessions, respectively.

### MEG measurements

A 160-channel MEG system (MEG vision; Yokogawa Electric Corporation, Tokyo, Japan) was used to assess the neural activity caused in the food and mosaic picture sessions. The MEG system has a magnetic field resolution of 4 fT/Hz^1/2^ in the white-noise region. The sensor and reference coils were gradiometers (diameter, 15.5 mm; baseline, 50 mm): The two coils were separated by 23 mm. The sampling rate was 1,000 Hz. The MEG data were high-pass filtered at 0.3 Hz.

### Spatial filtering analyses of the MEG data

Magnetic noise from outside the shielded room was eliminated by subtracting the data obtained from reference coils using specialized software (MEG 160; Yokogawa Electric Corporation, Tokyo, Japan). Parts of the MEG data which included artifacts were identified visually and excluded from analyses. The MEG data was analyzed using spatial filtering method to identify changes in oscillatory brain activity that reflected cortical activities induced by the food and mosaic pictures presented below the threshold of conscious awareness^24–26^. Sine alpha- and beta-band oscillatory brain activity has been reported to be related to cognitive control such as response inhibition^26,34^, MEG data were filtered at 8–13 Hz (i.e., alpha band) and 13–25 Hz (i.e., beta band) using a finite impulse response filtering method implemented in Brain Rhythmic Analysis for MEG software (BRAM; Yokogawa Electric Corporation, Tokyo, Japan). After the processing with the filtering methods, the estimation of the locations and intensities of neural activities were performed using BRAM, which employs a narrow-band adaptive spatial filtering algorithm^35,36^. Voxel size was set at 5.0 × 5.0 × 5.0 mm. Alpha- and beta-band powers of MEG data during viewing of visual stimuli in the food condition were calculated relative to those under the control condition (i.e., oscillatory power ratio) in 150-ms intervals from 0 to 1500 ms after the onset of mask-pictures.

### Group analyses of the MEG data

Group analyses of the data obtained from the spatial filtering analyses were performed using statistical parametric mapping software (SPM8; Wellcome Department of Cognitive Neurology, London, UK), implemented in Matlab 2011 (Mathworks, Natick, MA). The MR image of each participant was transformed into the Montreal Neurological Institute (MNI) T1-weighted image template^37^ and the parameter used in this transformation was applied to the data obtained from the spatial filtering analyses to normalize the MEG data. Smoothing of the anatomically normalized MEG data was performed using a Gaussian kernel of 20 mm (full-width at half-maximum) in the x-, y-, and z-axes. Individual data were incorporated into a random-effect model^38^ and the parameters estimated in the individual analysis was used to create “contrast” images and these “contrast” images were used for group analyses^38^. The significance in oscillatory band power between the food and control conditions (i.e., oscillatory power ratio) were assessed using *t* statistics (i.e., one-sample t test)^38^. The threshold for the one-sample t test was set at *P* < 0.005 (familywise-error corrected for multiple comparisons, FWE), considering the multiple comparison among the time windows and frequencies (i.e., alpha- and beta-band power). Localization of brain regions was performed using WFU_PickAtras, version 3.0.4 (http://fmri.wfubmc.edu/software/pickatlas) and Talairach Client, version 2.4.3 (http://www.talairach.org/client.html).

### Anatomical magnetic resonance (MR) imaging

MR images were obtained for each subject for generating subject-specific MEG source models. The images were obtained with a Philips Achieva 3.0 TX (Royal Philips Electronics, Eindhoven, the Netherlands). Five MRI compatible markers (Medtronic Surgical Navigation Technologies, Broomfield, CO) were placed on the head (i.e., two markers 10 mm in front of the left and right tragi, one marker 35 mm above the nasion, and two markers 40 mm to either side of the marker above the nasion). MEG data were co-registered to MR images using information obtained from these five markers and MEG localization coils.

### ECG

To examine changes in the measurements of ECG caused by viewing the food and mosaic pictures presented out of awareness, ECG was recorded during the eye-closed sessions just before and after the picture sessions in the food and control conditions using the EEG system (EEG-1518, Nihon Kohden, Tokyo, Japan) at a sampling rate of 1000 Hz. ECG data were transferred to the MEG system and analyzed with a maximum entropy method using MemCalc for Windows (Global Medical Solution, Tokyo, Japan). R-R wave variability was assessed: R-peaks extractions and the correction for ectopic beats were performed using MemCalc for windows. For frequency domain analysis of the R-R wave intervals, low-frequency power (LF) was calculated as the power within the frequency range of 0.04–0.15 Hz, and high-frequency power (HF) was calculated as that within the frequency range of 0.15–0.4 Hz. LF and HF were measured in absolute units (ms^2^). Since the unit for the R-R wave interval is millisecond (ms), the unit for the power spectral density is ms^2^/Hz. Therefore, the unit for LF and HF is ms^2^. HF has been reported as vagally mediated^39–41^, but LF originates from a variety of sympathetic and vagal mechanisms^39,42^. The LF/HF ratio has been considered to reflect sympathetic nervous system activity^43^. It is of note that there have been arguments about the interpretation of the origin of LF and LF/HF ratio in recent years^44^. However, since there have been reports that LH/HF ratio is related to pathophysiological states such as mental stress, fatigue, and so on^45–49^, LF/HF ratio seems to include important information about physiological processes. Therefore, there is a possibility that the alteration of LF/HF ratio is caused by the processes induced by the presentation of the masked food pictures. The natural logarithms of LF, HF, and LF/HF were calculated and used for statistical analyses (i.e., ln LF, ln HF, and ln LF/HF).

### Statistical analyses

Kolmogorov-Smirnov test was performed to confirm the normality of ln LF, ln HF, ln LF/HF, the score for cognitive restraint of food intake, and the increases of alpha band power observed in the BA 47 and BA13. Values are shown as mean and SD unless otherwise stated. A paired *t*-test with Bonferroni’s correction was used to compare the indices derived from the measurements of ECG such as LF and HF between food and control conditions. Relationships among increased alpha-band power, an index derived from the measurements of ECG, and a subscale of TFEQ were evaluated using Pearson’s correlation. Multivariate analysis of variance (MANOVA) for repeated measures was used to assess the effect of conditions (i.e., the food and control conditions) and time points (i.e., before and after the picture presentations) on the indices derived from the measurements of ECG (i.e., ln LF/HF, ln LF, and ln HF). All probability values were two-tailed and values of *P* < 0.05 were considered statistically significant. The statistical analyses mentioned above were performed using the SPSS version 21.0 software package (IBM, Armonk, NY).

## Results

### ECG analysis

**Figure 2:**
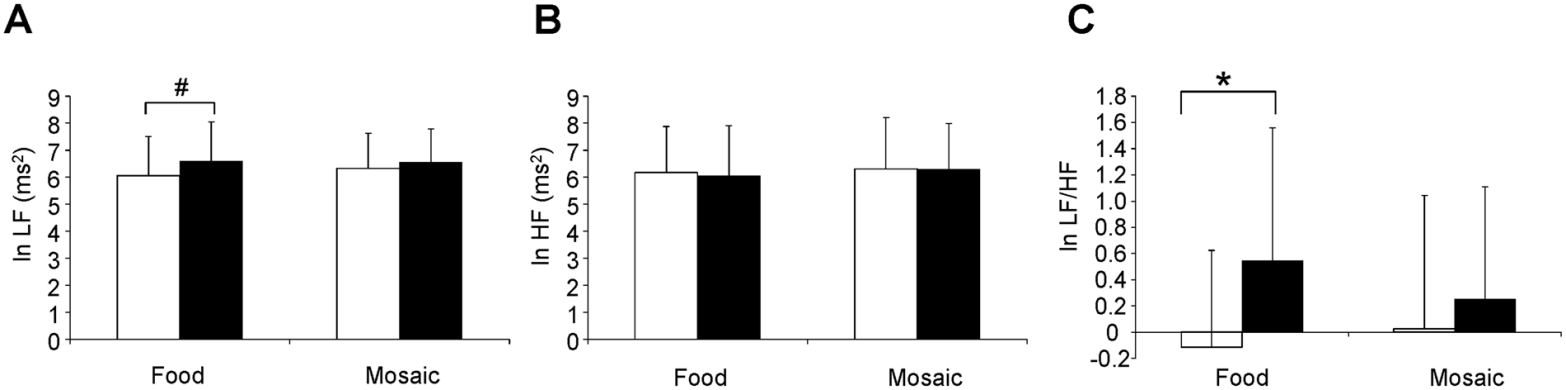
Alterations in the measurements of electrocardiography assessed by frequency domain analysis of the R-R wave intervals of electrocardiography are shown. Values were transformed by natural logarithm (ln). Low-frequency power (ln LF; **A**), high-frequency power (ln HF; **B**), and LF/HF ratio (ln LF/HF; **C**) in the food and control conditions are shown. White columns indicate values assessed just before the picture presentation and black columns indicate values assessed just after the picture presentation. While the ln LF/HF assessed just after the food picture session was increased compared with that assessed just before the session (*P* < 0.05, paired *t* test with Bonferroni’s correction), that assessed just after the mosaic picture session were not altered compared with that assessed just before the session. Data are presented as mean and SD. ^*^P < 0.05 and ^#^*P* < 0.10, paired *t*-test with Bonferroni’s correction.

**Figure 3:**
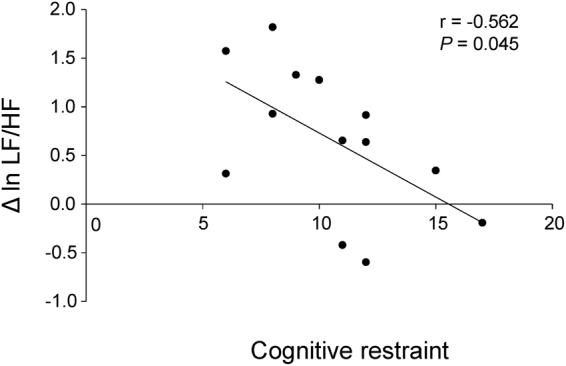
Relationship between increased LF/HF and level of cognitive restraint of food intake is sown. The increase in LF/HF during the presentation of food pictures in the food condition was negatively associated with the level of cognitive restraint of food intake. The linear regression line, Pearson’s correlation coefficient, and *P* value are shown.

Alterations in the measurements of ECG were analyzed for 13 participants, because 6 participants were excluded from analyses of MEG data as described below and ECG data from one participant whose MEG data were analyzed were not obtained due to a technical error of measurement. MANOVA for repeated measures which included three dependent variables (i.e., ln LF/HF, ln LF, and ln HF) was performed. The results showed that there was a main effect of time point (i.e., before and after the picture presentations) [F(2, 11) = 5.317, *P* = 0.024], but there was no main effect of conditions (i.e., the food and control conditions) [F(2, 11) = 1.205, *P* = 0.336] or conditions × time points interaction [F(2, 11) = 1.265, *P* = 0.320]. Since the number of the comparisons performed regarding the indices derived from the measurements of ECG were 6 (i.e., ln LF/HF, ln LF, and ln HF in the food and control conditions, respectively), the p-values for paired t test multiplied by 6 were reported below, to control the increase of a type I error caused by the multiple comparison (i.e., paired t test with Bonferroni’s correction). The ln LF assessed just after the food picture session showed an increasing tendency compared with that assessed just before the session (*P* < 0.10, paired *t* test with Bonferroni’s correction; Fig. 2). The ln LF/HF assessed just after the food picture session was increased compared with that assessed just before the session (*P* < 0.05, paired *t* test with Bonferroni’s correction; Fig. 2). The increase in ln LF/HF observed just after the food picture session correlated negatively with the level of cognitive restraint of food intake as assessed by TFEQ (r = −0.562, *P* = 0.045; Fig. 3). There was no correlation between the increment of ln LF/HF caused through the control condition and the level of cognitive restraint of food intake (r = −0.099, *P* = 0.748).

### Spatial filtering analyses of MEG data

MEG data from six participants were excluded from our analysis. MEG data from four participants were excluded because the participants reported that they recognized the food and/or mosaic pictures during MEG measurements. MEG data from the remaining two participants were contaminated with magnetic noise originating from outside the shielded room and the number of epochs that remained after exclusion of those that included artifacts was thus insufficient for analysis. We therefore analyzed MEG data from 14 participants: all 14 participants declared that they had not recognized food or mosaic pictures at any point during the experiment. The body mass index of these 14 participants was 21.1 ± 2.5 kg/m^2^.

**Figure 4:**
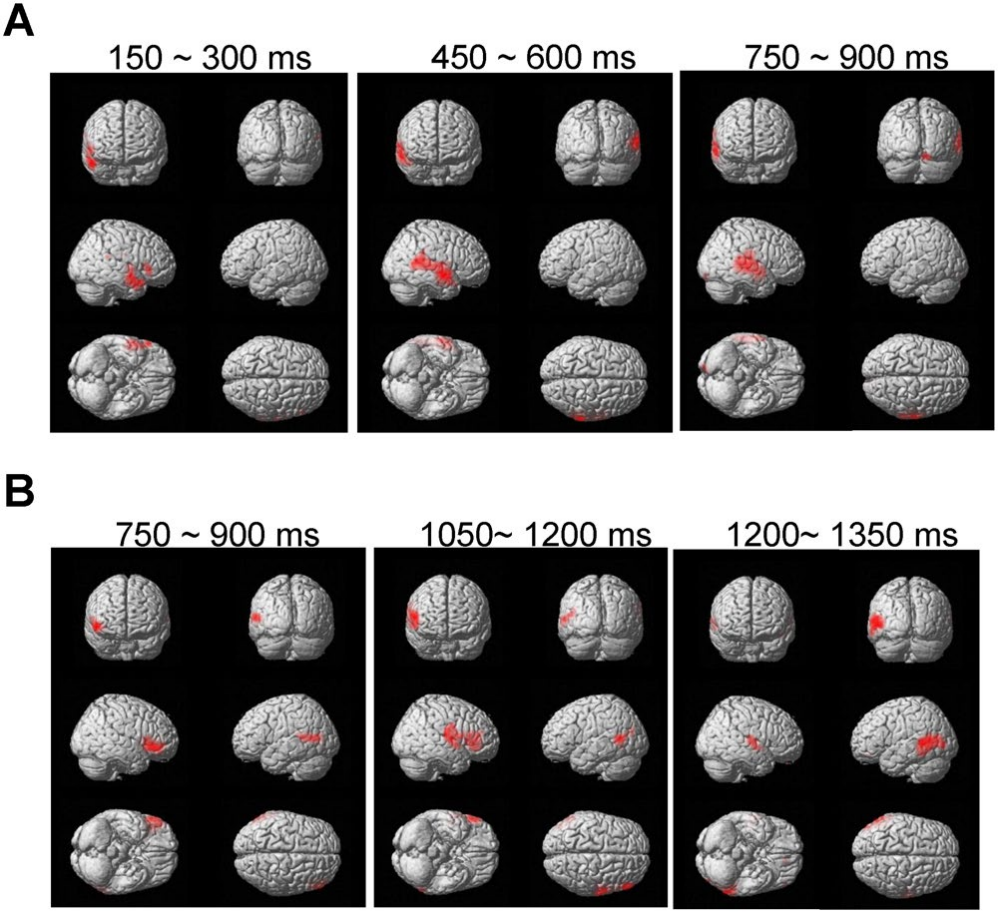
Statistical parametric maps of brain areas where alpha-band power was lower in the food condition than in the control conditions (**A**) and of brain areas where alpha-band power was higher in the food condition than in the control condition (**B**) are shown. Random-effect analyses of 14 participants, *P* < 0.05, familywise-error corrected for the entire search volumes.

To identify changes in neural activity caused by viewing food pictures presented below the threshold of awareness, the oscillatory powers observed while viewing the visual stimulus in the food condition were compared with those in the control condition. Brain regions were identified in which alpha-band power in the food condition was decreased (Fig. 4A) or increased (Fig. 4B) compared with that in the control condition (Table 1). The threshold for the SPM{t} of the one-sample t was set for P < 0.05 (FWE) for the purposes of presentation in Fig. 4.

**Table 1:** Brain regions showing greater decrease or increase in alpha band power in the food condition compared with the control condition.

Increase or decrease in oscillatory power relative to control condition	Time window	Location	BA	MNI coordinates (mm)			Z value
				x	y	z	
Decrease	150–300 ms	middle temporal gyrus	21	57	8	−30	4.4
	450–600 ms	middle temporal gyrus	21	67	−2	−10	4.3
	750–900 ms	superior temporal gyrus	42	62	−32	5	4.2
Increase	750–900 ms	inferior frontal gyrus	47	57	28	−5	4.5
		middle frontal gyrus	47	57	48	−5	4.4
		insula	13	47	8	5	3.8
	1050–1200 ms	precentral gyrus	6	62	3	10	4.8
		inferior frontal gyrus	45	57	33	5	4.1
		middle frontal gyrus	46	57	48	10	3.9
	1200–1350 ms	middle temporal gyrus	21	−63	−52	5	4.7
		middle occipital gyrus	19	−48	−82	5	3.8

BA, Brodmann’s area; MNI, Montreal Neurological Institute.

x, y, z: Stereotaxic coordinate.

Data were obtained from random-effect analyses. Only significant changes are shown (one sample t test, *P* < 0.005, familywise error rate).

**Figure 5:**
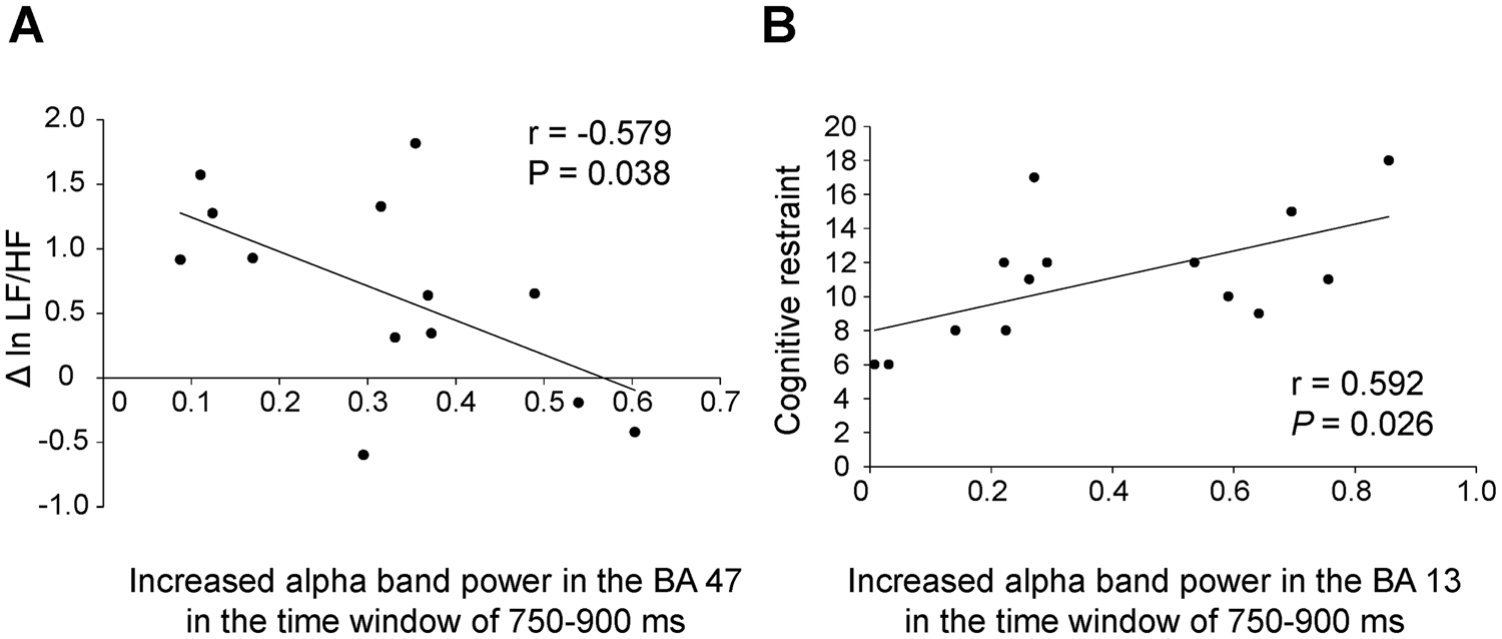
The increase in LF/HF during the presentation of food pictures in the food condition was negatively associated with the increased alpha-band power in BA47 observed in the food condition (**A**) and the level of cognitive restraint of food intake was positively associated with the increased alpha-band power in BA13 observed in the food condition (**B**). The linear regression line, Pearson’s correlation coefficient, and *P* value are shown.

The increase in alpha-band power in Brodmann area (BA) 47 in the time window of 750 to 900 ms after the onset of mask-pictures correlated negatively with the increase in ln LF/HF observed just after the food picture session (r = −0.579, *P* = 0.038; Fig. 5A). The increase in alpha-band power in BA13 in the time window of 750 to 900 ms after the onset of mask-pictures correlated positively with the level of cognitive restraint (r = 0.592, *P* = 0.026; Fig. 5B). There was no correlation between the increase in alpha-band power in BA 47 in the time window of 750 to 900 ms after the onset of mask-pictures and the increment of ln LF/HF caused through the control condition (r = −0.385, *P* = 0.193).

## Discussion

In the present study, participants viewed food and control pictures followed by mask pictures as food and control conditions, respectively. Among the 20 participants, 14 participants declared that they did not recognize any food or mosaic pictures during the experiment, and the data from these 14 participants were therefore analyzed. Only in the food condition, LF/HF assessed just after picture presentation was increased compared with that assessed just before presentation (i.e., the increase in LF/HF did not reach significance for the control condition), and this increase in LF/HF was negatively associated with the score for cognitive restraint. Increased and decreased alpha-band powers in several brain regions were observed under the food condition compared with the control condition (Table 1): The increased alpha-band power at BA47 in the time window of 750–900 ms after the onset of mask-pictures was negatively associated with the increase in LF/HF during the food condition and the increased alpha-band power at BA13 in the time window of 750–900 ms was positively associated with the score for cognitive restraint of food intake.

We used a visual backward masking procedure^19–21^ to present the visual food cue below the threshold of awareness. Since 14 of 20 participants declared that they had not recognized any food or mosaic pictures at all during the experiment, the food and control pictures were considered to have been successfully presented below the threshold of awareness for these 14 participants. LF/HF assessed just after the food picture session was increased compared with that assessed just before the session in these participants, even though they had not been consciously aware of the food pictures. This result shows that the presentation of masked food pictures had effects on the neural system despite being presented without the participant being aware^50^. However, although the alteration in LF/HF during the control conditions were not detected in our present study, we are not able to conclude that the increase of LF/HF observed in the food condition was greater than that in the control condition in our present study due to small sample size. In this sense, the findings regarding the alterations in LF/HF caused by the food and control conditions are preliminary (limitations of this work are described in more detail below). On the other hand, since the increase in LF/HF observed just after the food picture session correlated negatively with the level of cognitive restraint of food intake while there was no correlation between the increment of LF/HF caused through the control condition and the level of cognitive restraint of food intake, the interpretation of the increment of LF/HF caused in the food condition is different from that caused in the control condition (i.e., the increment of LF/HF in the food condition was related to the level of cognitive restraint of food intake although the increment of LF/HF in the control condition was not caused in relation to the level of cognitive restraint of food intake). Therefore, the finding that the LF/HF assessed just after the food picture session was increased compared with that assessed just before the session after the correction for multiple comparisons provides valuable clues to clarify the neural mechanisms related to appetitive behavior induced by the visual food cures presented below the threshold of awareness.

The increase in LF/HF observed in the food condition was negatively associated with the score for cognitive restraint of food intake as assessed by the TFEQ in participants who were not aware of the visual cue. Cognitive restraint is one of the cognitive and behavioral domains of eating behaviors and represents the conscious restriction of food intake to control body weight or promote weight loss^51^. This suggests that the individuals who engaged in higher levels of dietary restriction have lower levels of sympathetic tone when they view food pictures below the threshold of conscious awareness. However, contrary to our finding, there has been a report that individuals with restraint eating patterns showed elevations in heart rate in response to food pictures presented above the threshold of awareness^52^. In their study, since aversive response was also induced by food pictures in individuals with restraint eating patterns, activity of the sympathetic nervous system might have been modulated by subjective feelings of aversion when food pictures were presented in manner that the individual was aware of. As discussed in the next paragraph, taking that the negative correlation between the increased alpha-band power in BA 47 and the increased LF/HF during the food condition was observed in our present study, it may be that the individuals with high cognitive restraint of food intake suppressed the responses in ECG activity to visual food cues unconsciously. However, since there has been insufficient knowledge for the alterations in the responses in ECG activity caused by the presentation of food pictures either above or below the threshold of conscious awareness, further studies are needed in this point.

Several brain regions were seen in which alpha-band power was increased or decreased in the food condition compared with the control condition. Among these, the increased alpha-band power in BA47 (i.e., the inferior frontal gyrus) in the time window of 750–900 ms was negatively associated with the increased LF/HF seen in the food condition. A decrease or increase in oscillatory power in a specific frequency band has been reported as a specific feature of information processing^26^. Numerous reports have found that the right inferior frontal gyrus is involved in inhibitory control^53–60^ and it has been proposed that the increase of the alpha and beta band power is related to the inhibitory control^34,54,59^.Thus, the increase in alpha-band power observed in BA47 in our present study may reflect the inhibition of the responses to visual food cues presented below the threshold of awareness.

The increased alpha-band power in BA13 (i.e., the insular cortex) in the time window of 750–900 ms was positively associated with the score for cognitive restraint of food intake. In addition to serving as the taste cortex^61,62^, the insular cortex has been reported to be activated by visual food cues^9,14,63^, and activation is greater in obese individuals than in lean individuals^13,15^. Furthermore, activation in the insular cortex caused by visual food cues is reportedly related to subjective experience of appetite^64^ and to appetitive motives^9^. Taking these findings into consideration, the increase in alpha-band power observed in the insular cortex in the present study may indicate the suppression of appetite caused by food pictures presented below the threshold of awareness. This interpretation is in line with the observation that individuals with more increased alpha-band power in BA13 showed higher levels of cognitive restraint for food intake. In fact, the increase in alpha-band power has also been proposed to reflect deactivation of cortical areas involved in the processing of sensory or cognitive information^26,65^.

In the present study, changes to oscillatory neural activity in the inferior frontal gyrus and insular cortex were observed in the time window of 750–900 ms after the onset of mask images. However, in our previous study, the neural activity in the insular cortex caused by visual food pictures presented above the threshold of awareness was observed in the time window of 300 ms after the onset of pictures^9,10^. Since the findings in our present study were based on the enhancement of alpha band power and those in the previous study was based on the equivalent current dipole analysis, it may not adequate to discuss the difference in latency between these two studies. However, several speculations can be made on this point: This difference in latency may be due to the fact that the neural activity in the insular cortex observed in the present study (i.e., the increase of alpha band power in the insular cortex) was related to the suppression of appetite, while that observed in the previous study (i.e., the equivalent current dipole observed in the insular cortex) was related to the motivational aspect of appetite. In fact, neural activity related to the suppression of motivation to eat is reportedly related to decreased theta-band power in the DLPFC in the time window of 500–600 ms after the onset of presentation of food pictures^12^, suggesting that the immediate neural response to appetitive motives is followed by neural activity related to appetite suppression. Other explanations may be that the processing of information related to visual food cues presented below the threshold of awareness has a different time course from that related to visual food cues presented within the awareness of the individual, or that the neural processes related to visual food cues were interfered with by the backward masking procedure used in our present study.

There have been reports that the DLFPC is related to the suppression of appetitive motivation and dietary restraint^11,12^. However, the alteration of the neural activity in the DLPFC was not observed in our present study. The reason for this may be that the participants were not aware of the presentation of the food pictures and they were not instructed to intentionally suppress their appetite in our present study. It can be speculated that the neural activities related to the suppression of appetite caused by the food pictures presented above the threshold of awareness are different from those caused by the food pictures presented below the threshold of awareness.

Various limitations to this study need to be considered. First, the number of the participants was small. Although we successfully demonstrated that the neural activity induced by viewing a visual food cue presented below the threshold of awareness reflects appetitive behaviors in our present study, the statistical power for analyses regarding the indices derived from the measurements of ECG (i.e., ln LF, ln HF, and ln LF/HF) seems to be insufficient due to small number of subject. Therefore, we are not able to conclude that the increase of LF/HF observed in the food condition was greater than that observed in the control condition. Second, all participants in the present study were male. As eating disorders are more common in females than in males^66–68^, studies of female participants are likely to prove of great value in clarifying the pathophysiology of eating disorders. Third, subjective levels of appetite and/or appetitive motives under the food and control conditions were not assessed in the present study. We were thus unable to determine whether alteration of appetite and/or appetitive motives was caused by suppression of the insular cortex. We did not assess subjective levels of appetite and/or appetitive motives in the present study, as we wanted to avoid our participants knowing that the present experiment was related to appetite and visual food images. Fourth, we assessed the relationships among increased alpha-band power, an index of heart rate variability, and the level of cognitive restraint of food intake using a linear correlation coefficient. Therefore, it is difficult to determine the causality among variables from our present study.

In summary, the neural activity caused by visual food cues presented below the threshold of awareness was successfully evaluated using a backward masking procedure and MEG in the present study. This may show that neural activity induced by viewing a visual food cue presented below the threshold of awareness reflects appetitive behaviors, i.e., cognitive restraint of food intake. In addition, neural activity related to response inhibition and suppression of the brain region related to appetite and/or appetitive motives were observed in the individuals with high cognitive restraint of food intake when they view visual food cues below the threshold of awareness. Our findings may provide valuable clues to developing methods for assessing unconscious regulation of appetite in individuals with normal and abnormal eating behaviors, and may motivate further studies to clarify the neural mechanisms related to eating behaviors.

## References

[1] Ng M (2014). Global, regional, and national prevalence of overweight and obesity in children and adults during 1980-2013: a systematic analysis for the Global Burden of Disease Study 2013. Lancet.

[2] Wyatt SB, Winters KP, Dubbert PM (2006). Overweight and obesity: prevalence, consequences, and causes of a growing public health problem. The American journal of the medical sciences.

[3] Rosenbaum M, Leibel RL, Hirsch J (1997). Obesity. N Engl J Med.

[4] Hagan S, Niswender KD (2012). Neuroendocrine regulation of food intake. Pediatric blood & cancer.

[5] Berthoud HR (2004). Neural control of appetite: cross-talk between homeostatic and non-homeostatic systems. Appetite.

[6] Berthoud HR (2006). Homeostatic and non-homeostatic pathways involved in the control of food intake and energy balance. Obesity (Silver Spring).

[7] Suzuki K, Jayasena CN, Bloom SR (2012). Obesity and appetite control. Experimental diabetes research.

[8] Pursey KM (2014). Neural responses to visual food cues according to weight status: a systematic review of functional magnetic resonance imaging studies. Frontiers in nutrition.

[9] Yoshikawa T, Tanaka M, Ishii A, Watanabe Y (2013). Immediate neural responses of appetitive motives and its relationship with hedonic appetite and body weight as revealed by magnetoencephalography. Medical science monitor: international medical journal of experimental and clinical research.

[10] Yoshikawa T, Tanaka M, Ishii A, Watanabe Y (2014). Suppressive responses by visual food cues in postprandial activities of insular cortex as revealed by magnetoencephalography. Brain Res.

[11] Hollmann M (2012). Neural correlates of the volitional regulation of the desire for food. Int J Obes (Lond).

[12] Yoshikawa T, Tanaka M, Ishii A, Fujimoto S, Watanabe Y (2014). Neural regulatory mechanism of desire for food: revealed by magnetoencephalography. Brain Res.

[13] Stice E, Spoor S, Bohon C, Veldhuizen MG, Small DM (2008). Relation of reward from food intake and anticipated food intake to obesity: a functional magnetic resonance imaging study. Journal of abnormal psychology.

[14] Schienle A, Schafer A, Hermann A, Vaitl D (2009). Binge-eating disorder: reward sensitivity and brain activation to images of food. Biol Psychiatry.

[15] Scharmuller W, Ubel S, Ebner F, Schienle A (2012). Appetite regulation during food cue exposure: a comparison of normal-weight and obese women. Neurosci Lett.

[16] Forman EM (2017). Promising technological innovations in cognitive training to treat eating-related behavior. Appetite.

[17] Finlayson G, King N, Blundell J (2008). The role of implicit wanting in relation to explicit liking and wanting for food: implications for appetite control. Appetite.

[18] Munzberg H, Qualls-Creekmore E, Yu S, Morrison CD, Berthoud HR (2016). Hedonics Act in Unison with the Homeostatic System to Unconsciously Control Body Weight. Frontiers in nutrition.

[19] Breitmeyer BG, Ogmen H (2000). Recent models and findings in visual backward masking: a comparison, review, and update. Perception & psychophysics.

[20] Enns JT, Di Lollo V (2000). What’s new in visual masking?. Trends Cogn Sci.

[21] Noguchi Y, Kakigi R (2005). Neural mechanisms of visual backward masking revealed by high temporal resolution imaging of human brain. Neuroimage.

[22] Esteves F, Arriaga P, Carneiro P, Flykt A (2010). Emotional responses (verbal and psychophysiological) to pictures of food stimulr. Psicologia.

[23] Uher R, Brooks SJ, Bartholdy S, Tchanturia K, Campbell IC (2014). Increasing cognitive load reduces interference from masked appetitive and aversive but not neutral stimuli. PLoS One.

[24] David, O., Kilner, J. M. & Friston, K. J. Mechanisms of evoked and induced responses in MEG/EEG. *Neuroimage* **31**, 1580–1591; S1053-8119(06)00117-0 [pii] 10.1016/j.neuroimage.2006.02.034 (2006). 10.1016/j.neuroimage.2006.02.034 16632378

[25] Hillebrand A, Singh KD, Holliday IE, Furlong PL, Barnes GR (2005). A new approach to neuroimaging with magnetoencephalography. Hum Brain Mapp.

[26] Pfurtscheller G, Lopes da Silva FH (1999). Event-related EEG/MEG synchronization and desynchronization: basic principles. Clin Neurophysiol.

[27] Oldfield RC (1971). The assessment and analysis of handedness: the Edinburgh inventory. Neuropsychologia.

[28] Nutrition, K. E. I. o. *Mainichi no Shokuji no Karori Gaido (in Japanese)*. 11–150 (Kagawa Nutrition University Publishing Division, 2008).

[29] Nakamura K (2000). Functional delineation of the human occipito-temporal areas related to face and scene processing. A PET study. Brain.

[30] Allison T, McCarthy G, Nobre A, Puce A, Belger A (1994). Human extrastriate visual cortex and the perception of faces, words, numbers, and colors. Cereb Cortex.

[31] Stunkard AJ, Messick S (1985). The three-factor eating questionnaire to measure dietary restraint, disinhibition and hunger. J Psychosom Res.

[32] Karlsson J, Persson LO, Sjostrom L, Sullivan M (2000). Psychometric properties and factor structure of the Three-Factor Eating Questionnaire (TFEQ) in obese men and women. Results from the Swedish Obese Subjects (SOS) study. International journal of obesity and related metabolic disorders: journal of the International Association for the Study of Obesity.

[33] Cappelleri JC (2009). Psychometric analysis of the Three-Factor Eating Questionnaire-R21: results from a large diverse sample of obese and non-obese participants. Int J Obes (Lond).

[34] Klimesch W, Sauseng P, Hanslmayr S (2007). EEG alpha oscillations: the inhibition-timing hypothesis. Brain Res Rev.

[35] Dalal SS (2008). Five-dimensional neuroimaging: localization of the time-frequency dynamics of cortical activity. Neuroimage.

[36] Sekihara, K. & Nagarajan, S. S. *Adaptive Spatial Filters for Electromagnetic Brain Imaging*. 1–203 (Springer Verlag, 2008).

[37] Evans, A. C., Kamber, M., Collins, D. L. & MacDonald, D. In *Magnetic resonance scanning and epilepsy* (eds S. D. Shorvon *et al*.) 263-274 (Plenum Press, 1994).

[38] Friston KJ, Holmes AP, Worsley KJ (1999). How many subjects constitute a study?. Neuroimage.

[39] Akselrod S (1981). Power spectrum analysis of heart rate fluctuation: a quantitative probe of beat-to-beat cardiovascular control. Science.

[40] Pomeranz B (1985). Assessment of autonomic function in humans by heart rate spectral analysis. Am J Physiol.

[41] Malliani A, Pagani M, Lombardi F, Cerutti S (1991). Cardiovascular neural regulation explored in the frequency domain. Circulation.

[42] Appel ML, Berger RD, Saul JP, Smith JM, Cohen RJ (1989). Beat to beat variability in cardiovascular variables: noise or music?. Journal of the American College of Cardiology.

[43] Pagani M (1997). Relationship between spectral components of cardiovascular variabilities and direct measures of muscle sympathetic nerve activity in humans. Circulation.

[44] Reyes del Paso GA, Langewitz W, Mulder LJ, van Roon A, Duschek S (2013). The utility of low frequency heart rate variability as an index of sympathetic cardiac tone: a review with emphasis on a reanalysis of previous studies. Psychophysiology.

[45] Hjortskov N (2004). The effect of mental stress on heart rate variability and blood pressure during computer work. European journal of applied physiology.

[46] Seong HM, Lee JS, Shin TM, Kim WS, Yoon YR (2004). The analysis of mental stress using time-frequency distribution of heart rate variability signal. Conference proceedings:… Annual International Conference of the IEEE Engineering in Medicine and Biology Society. IEEE Engineering in Medicine and Biology Society. Conference.

[47] Tanaka M (2011). Autonomic nervous alterations associated with daily level of fatigue. Behavioral and brain functions: BBF.

[48] Mizuno K (2011). Mental fatigue caused by prolonged cognitive load associated with sympathetic hyperactivity. Behavioral and brain functions: BBF.

[49] Kemp AH, Quintana DS, Felmingham KL, Matthews S, Jelinek HF (2012). Depression, comorbid anxiety disorders, and heart rate variability in physically healthy, unmedicated patients: implications for cardiovascular risk. PLoS One.

[50] Critchley, H. D., Eccles, J. & Garfinkle, S. In *Handbook of Clinical Neurology* Vol. 117 (eds R. M. Buijs & D. F. Swaab) Ch. 6, 59–77 (Elsevier, 2013). 10.1016/B978-0-444-53491-0.00006-7 24095116

[51] Cappelleri JC (2009). Evaluating the Power of Food Scale in obese subjects and a general sample of individuals: development and measurement properties. Int J Obes (Lond).

[52] Drobes DJ (2001). Food deprivation and emotional reactions to food cues: implications for eating disorders. Biological psychology.

[53] Garavan H, Ross TJ, Stein EA (1999). Right hemispheric dominance of inhibitory control: an event-related functional MRI study. Proc Natl Acad Sci USA.

[54] Swann N (2009). Intracranial EEG reveals a time- and frequency-specific role for the right inferior frontal gyrus and primary motor cortex in stopping initiated responses. J Neurosci.

[55] Chambers CD (2007). Dissociable mechanisms of cognitive control in prefrontal and premotor cortex. J Neurophysiol.

[56] Neubert FX, Mars RB, Buch ER, Olivier E, Rushworth MF (2010). Cortical and subcortical interactions during action reprogramming and their related white matter pathways. Proc Natl Acad Sci USA.

[57] Jacobson L, Javitt DC, Lavidor M (2011). Activation of inhibition: diminishing impulsive behavior by direct current stimulation over the inferior frontal gyrus. Journal of cognitive neuroscience.

[58] Zanto TP, Rubens MT, Thangavel A, Gazzaley A (2011). Causal role of the prefrontal cortex in top-down modulation of visual processing and working memory. Nat Neurosci.

[59] Swann NC (2012). Roles for the pre-supplementary motor area and the right inferior frontal gyrus in stopping action: electrophysiological responses and functional and structural connectivity. Neuroimage.

[60] Sacchet MD (2015). Attention drives synchronization of alpha and beta rhythms between right inferior frontal and primary sensory neocortex. J Neurosci.

[61] Kurth F, Zilles K, Fox PT, Laird AR, Eickhoff SB (2010). A link between the systems: functional differentiation and integration within the human insula revealed by meta-analysis. Brain structure & function.

[62] Small DM (2010). Taste representation in the human insula. Brain structure & function.

[63] Huerta CI, Sarkar PR, Duong TQ, Laird AR, Fox PT (2014). Neural bases of food perception: coordinate-based meta-analyses of neuroimaging studies in multiple modalities. Obesity (Silver Spring).

[64] Porubska K, Veit R, Preissl H, Fritsche A, Birbaumer N (2006). Subjective feeling of appetite modulates brain activity: an fMRI study. Neuroimage.

[65] Pfurtscheller G (1992). Event-related synchronization (ERS): an electrophysiological correlate of cortical areas at rest. Electroencephalogr Clin Neurophysiol.

[66] Hudson JI, Hiripi E, Pope HG, Kessler RC (2007). The prevalence and correlates of eating disorders in the National Comorbidity Survey Replication. Biol Psychiatry.

[67] Hoek HW, van Hoeken D (2003). Review of the prevalence and incidence of eating disorders. The International journal of eating disorders.

[68] Swanson SA, Crow SJ, Le Grange D, Swendsen J, Merikangas KR (2011). Prevalence and correlates of eating disorders in adolescents. Results from the national comorbidity survey replication adolescent supplement. Arch Gen Psychiatry.

